# Regional and social differences concerning overweight, participation in health check-ups and vaccination. Analysis of data from a whole birth cohort of 6-year old children in a prosperous German city

**DOI:** 10.1186/1471-2458-9-43

**Published:** 2009-01-30

**Authors:** Daniela Koller, Andreas Mielck

**Affiliations:** 1 University of Bremen, Centre for Social Policy Research, Division Health Economics, Health Policy and Outcomes Research, Bremen, Germany; 2 Helmholtz Zentrum Muenchen – German Research Center for Environmental Health, Institute of Health Economics and Health Care Management, 85758 Neuherberg, Germany

## Abstract

**Background:**

Studies on health inequalities still focus mostly on adults. Research about social disparities and health in children is slowly increasing, also in Germany, but these studies are mostly restricted to individual social variables derived from the parents to determine social class. This paper analyses the data of the medical check-up prior to school enrolment to determine differences concerning overweight, participation in health check-ups and immunization; it includes individual social variables but also regional variables describing the social environment of the children.

**Methods:**

The dataset includes 9,353 children who started school in 2004 in Munich, Germany. Three dependent variables are included (i.e. overweight, health check-ups, vaccinations). The individual level social variables are: children's sex, mother tongue of the parents, Kindergarten visit. On the small scale school district level, two regional social variables could be included as well, i.e. percentage of single-parent households, percentage of households with low educational level. Associations are assessed by cross tables and regression analyses. The regional level variables are included by multilevel analyses.

**Results:**

The analyses indicate that there is a large variation between the school districts concerning the three dependent variables, and that there is no district with very 'problematic values' for all three of them (i.e. high percentage of overweight, low levels of health check-ups and vaccinations). Throughout the bivariate and multivariate analyses, the mother tongue of the children's parents shows the most pronounced association with these dependent variables; i.e. children growing up in non-German-speaking families tend to be more overweight and don't visit preventive check-ups as often as children of German-speaking parents. An opposite association can be seen concerning vaccinations. Regional level influences are present as well, but they are rather small when the individual level social variables are controlled for.

**Conclusion:**

The dataset of the medical check-up prior to school enrolment offers a great opportunity for public health research, as it comprises a whole age cohort. The number and scope of variables is quite limited, though. On one hand, it includes only few variables on health or health related risks. On the other, it would be important to have more information from the region where the children live, e.g. the availability of community and health care services for parents and children, social networks of families with children, areas where children can play outside, traffic noise and air pollution. Despite these shortcomings, the need for specific interventions can already be derived from the data analyzed here, e.g. programs to reduce overweight in children should focus on parents with a mother tongue other than German.

## Background

In the past few years, health inequalities have been in the centre of public health research, but most studies still focus on adults [1]. This is also true for Germany [2]. Recently, health inequalities among children have gained increased attention [3,4]. In Germany, this increased interest has been supported by two large empirical studies: One is named 'Health Behaviour in School-Aged Children (HBSC)', a "cross-national research study conducted in collaboration with the WHO Regional Office for Europe" . In order to assess the social status of the family, a "Family Affluence Scale" has been developed, and the analyses clearly show that the lowest status group has the highest prevalence of poor health and of risk factors such as obesity and smoking [5]. The results published by the German study team  confirm that very similar associations can be seen in the Germany sample (n = 5,650) as well [6]. The other study is called 'German Health Interview and Examination Survey for Children and Adolescents' (KIGGS). "The KiGGS study was designed as a comprehensive, nation-wide, representative interview and examination survey for the age group 0–17 years. Between May 2003 and May 2006, a total of 17,641 participants from 167 communities were enrolled." . The analyses again show that poor health and risk factors such as obesity and smoking are most prevalent in the lowest status group [7].

In these studies, as in most studies on social inequalities in children health, social status is assessed by individual level factors such as income of the parents or family car ownership [3,4,8-12]. This is even true for studies focussing on environmental factors such as air pollution, traffic noise and housing conditions [13]. There are very few analyses looking at the association between regional deprivation and child health [14,15], although these studies would be important, as regional level risk factors could have an independent effect over and above individual level risk factors [16], and as they could strengthen the setting approach by focussing on small regions.

The small number of regional studies concerning health inequalities in Germany is quite surprising, as regional differences in health are known to be important. Most studies on regional factors have been conducted in the USA and the United Kingdom, though. In a paper by Joshi et al. it is stated, for example, that regional differences in health are an important public health issue in Great Britain at least since the nineteenth century, when William Farr established 'healthy districts'. They also stress that these regional differences persisted and that they mirror disparities in social structure and also in individual behaviour [17]. The regional distribution of health, health risks and social characteristics is discussed now in many publications [18-20]. Few papers focus on children, but most probably regional differences can be found among children as well. It can even be argued that regional differences are especially pronounced among children, as they spend most of their time in their local neighbourhood. Children are strongly influenced by the health behaviour of their parents of course [21], and focussing on children could thus also be a good chance to study the interplay between regional and individual risk factors.

In Germany, the medical check-up prior to school enrolment offers a very good opportunity to analyse health problems of young children and factors influencing it. It includes the whole population of the age-cohort, and it also gives a chance for small scale regional comparisons. In the German school system, primary schools are organized by school districts. The children are obliged to visit the school of their district, giving the chance to examine inner-community differences of health and social factors on a very small regional scale. Based on the data from the City of Munich, Germany, the present analysis aims at answering the following question: Are there social and regional differences concerning overweight, participation in health check-ups and immunization among children in the city of Munich?

## Methods

The main basis for the analyses are the data from the medical check-up of the cohort starting school in 2004, provided by the Department for Health and Environment of the City of Munich, Germany. The city of Munich was chosen for several reasons: The results from the medical check-ups are available in a data set (which is not the case for all communities in Germany); some information on the social structure is available per school district (which is rarely the case in Germany); Munich is a prosperous city and public awareness of health inequalities is rather low. The data set includes the whole age cohort; every child has to be presented to a school nurse before starting school. If any health problems are observed by the nurse, a school physician is consulted as well [22].

For the school year starting in 2004, a total of 9,883 children were presented to the school nurse [23]. We excluded those children who could not be clearly linked to a school district (missing or wrong school district number). Children signing in for the German-French school were excluded too because this school (being a private school) does not have its own school district. Finally, we could include 9,353 children (94.6%).

The dataset includes just a few variables on health related factors for each child. We focussed our analyses on those that were assessed in a rather objective way: overweight (defined by the Body-Mass-Index, BMI), participation in preventive health check-ups for children (in Germany called 'U1 to U9'), and vaccinations. Other indicators included in the check-up (such as impaired vision or hearing disorders) were disregarded, as the number of cases per school district is very low, and as they were assessed on a rather imprecise way.

For adults, overweight is commonly defined by a simple BMI value such as 25 [24]. For children, age- and sex-specific limits should be used. For Germany, these limits have been defined by Kromeyer-Hausschild et al. [25], based on several studies conducted between 1985 and 1999 in different German regions. We have chosen this definition as the study focuses on children living in Germany. We have also conducted additional analyses based on a definition that is more common in the international literature, i.e. the definition proposed by Cole et al. [26]. The results were very similar, though, and are not reported below. The limit for overweight is defined as '90% or above' the age and sex specific distribution. We applied this limit and defined two groups (i.e. normal weight, overweight). Body height and weight were measured during the medical check-up, probably yielding reliable data.

The nine preventive health check-ups 'U1 to U9' are offered to every child between birth and six years of age. The aim is to identify any health problem of the child, thus giving some information on the 'health awareness' of the parents. The costs are covered by the statutory health insurance, i.e. there are no extra out of pocket payments for the parents. It is strongly recommended to visit all nine check-ups; the consultations are documented in the so called 'yellow booklet'. This information can be included here, as the parents are asked to show this booklet to the school nurse. It is assumed that parents who presented their children to all check-ups are more concerned about the health of their children than other parents. Two groups are differentiated for the analyses: a) children visiting all nine preventive check-ups; b) children missing at least one of these check-ups.

Concerning vaccinations, we focused only on those recommended by the German 'permanent vaccination commission' of the Robert Koch Institute, i.e. measles, mumps, rubella, diphtheria, hepatitis B, meningitis, tetanus, pertussis, polio [27]. Children having received all nine vaccinations are categorized as 'completely immunized'. If one or more vaccination is missing, the child is placed in the category 'not completely immunized'.

These three dependent variables are combined with social variables on the individual and on the regional level. On the individual level, the dataset includes:

- sex of the child (boy/girl)

- mother tongue of the parent

- child had visited the Kindergarten for at least twelve months (yes/no)

The mother tongue of the parents is coded for the mother and the father. It is not further specified if this language is the only spoken language by the parent but it can be seen as a more reliable indicator for the migration background than the indicator 'nationality', as having a German passport does not necessarily imply good understanding of the German language. For this analysis we distinguished two groups of children: both parents 'German language', at least one parent 'other language'.

Concerning the school districts level, data for two variables could be acquired from the Statistical Office of the City of Munich; they can be understood as surrogates for variables not available at the individual level:

- percentage of single-parent-households (grouped into tertiles: low, medium, high percentage)

- percentage of households with at least one adult having the lowest educational level (grouped into tertiles: low, medium, high percentage)

The German school system differentiates between three main educational levels. The lowest is the 'Hauptschulabschluss' which is usually gained after nine years of school education. The next is the 'Mittlere Reife' and the highest is the 'Abitur'.

The statistical analyses were calculated with the SAS software (Version 9.1). To identify associations between the dependent and independent variables, we first calculated cross tables (including chi^2^-tests). Then we performed the following multivariate analyses: For each of the three dependent variables logistic regressions were calculated. The first model only includes the individual social variables (i.e. sex of the child, mother tongue of parents, Kindergarten visit of the child), and the second model only includes the regional social variables (i.e. percentage of single-parent-households, of households with lower educational level). In a third step, all independent variables were included simultaneously in a multilevel-analysis (on 'level 1' the three individual social variables, and on 'level 2' the two regional variables). A multilevel analysis is necessary for controlling auto-correlations when the data can be split into different 'levels' [28]. The statistical analysis was performed with SAS using the glimmix procedure (with random intercept). In order to gain results that can more easily be communicated to policy makers, we performed a separate analysis for each of the two 'level 2' variables. In addition, the regional distribution is visualized by including a thematic map, produced with the ESRI software ArcGIS (Version 9.1).

## Results

Table 1 shows the distribution of the dependent variables. Missing values are quite rare for overweight, and for the other two dependent variables they are still lower than 9%. The prevalence of overweight (10.29%) is much lower than for incomplete check-ups (23.15%) and for incomplete immunizations (55.14%). The comparison between the 125 school districts shows large discrepancies. Incomplete check-ups, for example, range between 6.9 and 45.8 percent.

**Table 1:** Distribution of the dependent variables

	over-weight^a^	incomplete U1-U9 check-ups^b^	incomplete immunizations^c^
missing values (%)^d^	286 (3.05%)	839 (8.97%)	785 (8.39%)

individual level^e^			
- n (%)	933 (10.29%)	1.971 (23.15%)	4.724 (55.14%)
school district level^f^			
- mean	10.18%	22.83%	55.73%
- minimum	2.17%	6.94%	35.19%
- maximum	25.71%	45.76%	80.00%

a) BMI ≥ 90. percentile

b) at least one of the nine check-ups missing

c) at least one out of the nine vaccinations missing

d) total n = 9.353 children (first grader 2004 in Munich)

e) distribution without taking the school districts into account

f) distribution per school district (total n = 125 school districts)

**Figure 1:**
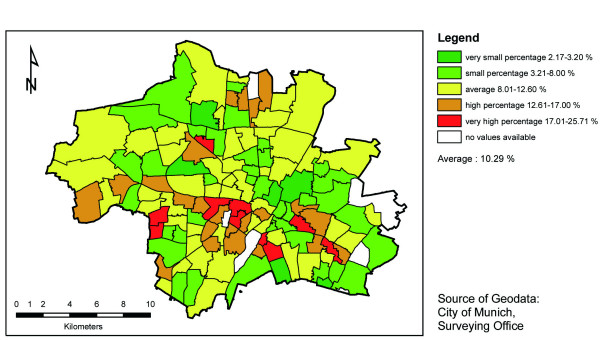
**Prevalence of overweight (BMI ≥ 90. Percentile) in school districts in Munich**.

The graphical visualization shows that the distribution across the school districts is quite different for each dependent variable. No school district could be identified with a particularly high (resp. low) percentage in all three variables (i.e. overweight, incomplete check-ups or incomplete immunization). For illustration, the distribution for overweight is shown in figure 1. There seems to be no clear regional concentration within the city. In the 'best' school districts only 2.17 percent of the children are overweight, and in the 'worst' this percentage is as high as 25.71 percent. As can be seen on the map, high rates of overweight are found for example in the centre. These city quarters are known to be characterised by a relatively high level of social deprivation, and by few places for children to play outside due to a high building density [29].

**Figure 2:**
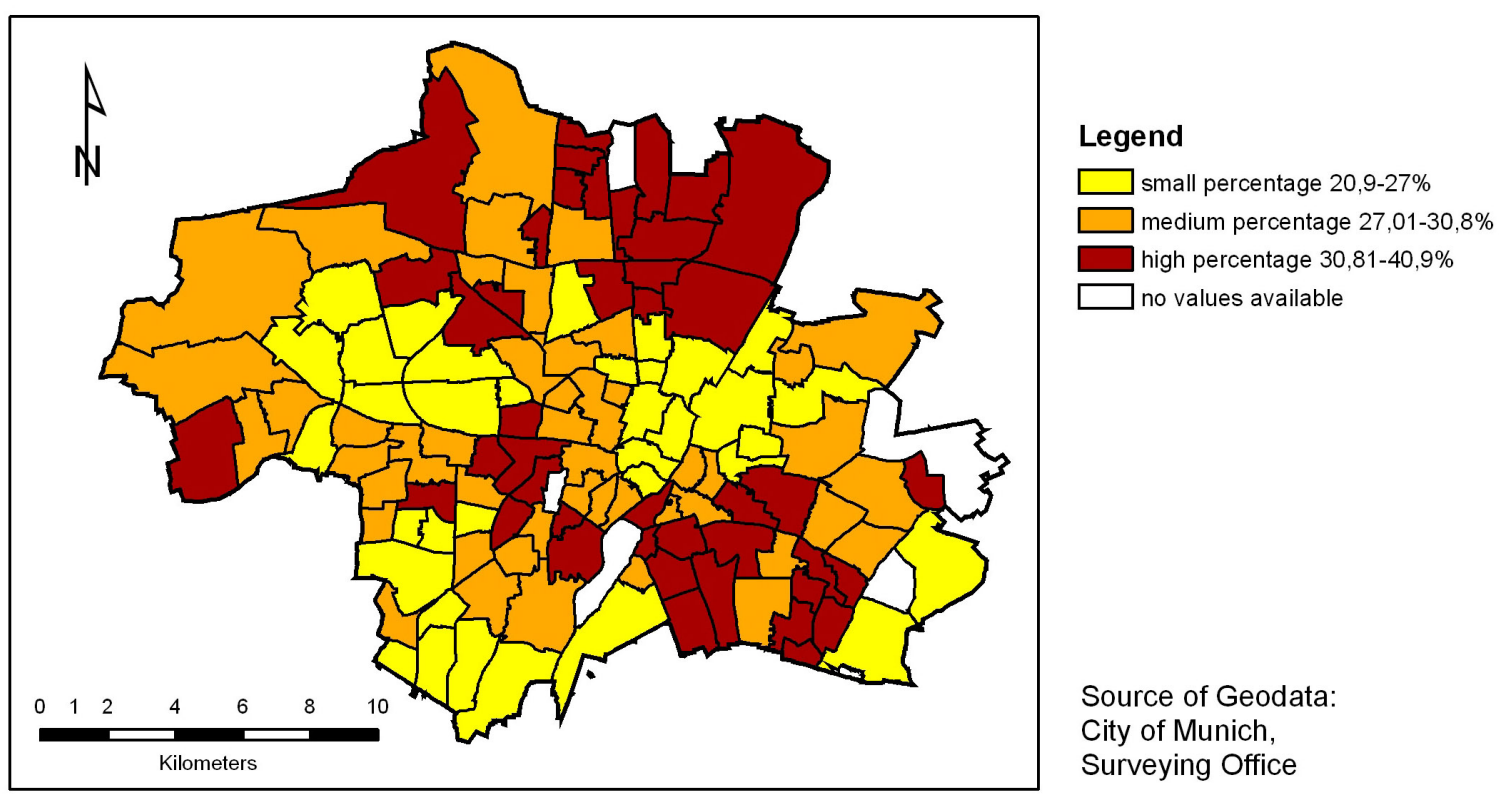
**Percentage of households with low education in school districts in Munich *(see 'methods' for definition of 'low education')***.

In order to illustrate the regional distribution of an independent variable, the distribution of low education households is shown in figure 2. The areas showing high values (especially in the northern and in the south-eastern part of Munich) are known to be characterised by high concentrations of people with low social status. The visual comparison between the two maps indicates that the distribution of overweight does not directly follow the distribution of low education households, that the association is rather complex and should be assessed in multivariate analyses.

The results of the bivariate associations are shown in table 2. Some differences are particularly large. Concerning overweight, for example, the prevalence is 6.71% for children of parents with German mother tongue, and 17.33% for children of parents with another mother tongue. A similar association can also be seen between incomplete check-ups and mother tongue of the parents. Concerning incomplete immunizations, though, an opposite association emerges (i.e. the prevalence is somewhat higher in the group 'German mother tongue' than in the group 'other mother tongue'). It can also be pointed out that children visiting a Kindergarten for at least 12 months show a lower prevalence of overweight and of incomplete health check-ups (as compared with children without this Kindergarten visit). Looking at the regional level, school districts characterized by a high percentage of single parent households and 'low education households' also have the highest percentage of children with overweight and incomplete health check-ups. These differences are not as large as the differences on the individual level, but they reveal a dose-response pattern and they are statistically significant. For the dependent variable 'incomplete immunizations', though, the opposite association is found. Also, there is a significant association (p < 0.001; chi^2^-test) between the two independent variables on the school district-level. A significant association can also be seen for the variables on the individual level, i.e. between the variable 'mother tongue of the parents' on one hand and 'visit to Kindergarten' on the other.

**Table 2:** Bivariate associations between the dependent and independent variables

	over-weight^a^		incomplete U1-U9 check-ups^b^		incomplete immunizations^c^	
	n	*%*	n	*%*	n	*%*
individual level						

mother tongue of parents^d^						
- German	384	6.71	904	16.18	3.243	58.93
- other	513	17.33	979	37.61	1.292	47.22
p-value^e^		<0.0001		<0.0001		<0.0001
visit to Kindergarten^f^						
- yes	815	9.89	1.737	21.99	4.329	54.96
- no	86	15.36	186	44.60	277	57.59
p-value^e^		<0.0001		<0.0001		0.2601
sex of the child						
- boy	483	10.37	962	22.41	2.367	54.94
- girl	450	10.21	992	24.00	2.311	55.45
p-value^e^		0.8041		0.0832		0.6422

school district level						

single parent households^g^						
- low (1.8 – 3.0%)	240	8.95	519	20.56	1.500	59.06
- medium (3.1 – 3.7%)	329	9.93	651	20.86	1.756	55.98
- high (3.8 – 8.4%)	364	11.84	801	27.91	1.468	50.78
p-value^e^		0.0011		<0.0001		<0.0001
'low education households'^h^						
- low (20.9 – 27.0%)	173	7.26	408	18.09	1.334	59.34
- medium (27.1 – 30.8%)	315	9.97	637	21.35	1.707	56.96
- high (30.9 – 40.9%)	445	12.62	926	28.27	1.683	50.65
p-value^e^		<0.0001		<0.0001		<0.0001

a) BMI ≥ 90. percentile

b) at least one of the nine check-ups missing

c) at least one out of the nine vaccinations missing

d) German: both parents with mother tongue German; other: all other parents

e) χ^2^-test

f) yes: child visited Kindergarten for at least one year; no: all other children

g) percentage of single parent households

h) percentage of persons having the lowest educational level (i.e. 'Hauptschulabschluss')

The results of the multivariate logistic regression again show the strong associations with the individual level variable 'mother tongue of the parents' (see table 3): In the group 'non German mother tongue', the risk of overweight is about three times as high as in the group 'German mother tongue'. A similar association can also be found concerning incomplete health check-ups. The dependent variable 'incomplete immunizations' again shows the opposite association. A Kindergarten visit is not any more associated with overweight (as compared with table 1), but children without this experience clearly show increased risks for incomplete health check-ups and for incomplete immunizations. Looking at the regional level, the largest associations are found for the variable 'percentage of low education households', with significant odds ratios showing a dose-response pattern.

**Table 3:** Multivariate Analysis (logistic regression)

	overweight^a^		incomplete U1-U9 check-ups^b^		incomplete immunizations^c^	
	OR	95% CI	OR	95% CI	OR	95% CI
individual level*						

Mother tongue parents^d^						
- German	1.00		1.00		1.00	
- other	***2.96***	***2.56–3.42***	***2.94***	***2.63–3.28***	***0.60***	***0.55–0.66***
Kindergarten visit of the child^e^						
- yes	1.00		1.00		1.00	
- no	1.12	0.87–1.44	***2.07***	***1.67–2.56***	***1.31***	***1.07–1.59***
Sex of the child						
- boy	1.00		1.00		1.00	
- girl	0.99	0.86–1.14	1.10	0.99–1.22	1.00	0.91–1.09

school district level**						

percentage of						
single parent households^f^						
- low (1.8 – 3.0%)	1.00		1.00		1.00	
- medium (3.1 – 3.7%)	1.02	0.85–1.22	0.93	0.82–1.07	0.92	0.83–1.03
- high (3.8 – 8.4%)	1.04	0.84–1.27	***1.16***	***1.00–1.34***	***0.83***	***0.73–0.94***
'low education households'^g^						
- low (20.9 – 27.0%)	1.00		1.00		1.00	
- medium (27.1 – 30.8%)	***1.41***	***1.16–1.71***	***1.21***	***1.06–1.39***	0.92	0.82–1.03
- high (30.9 – 40.9%)	***1.81***	***1.47–2.24***	***1.63***	***1.40–1.90***	***0.77***	***0.68–0.88***

*) simultaneous control of: mother tongue, Kindergarten visit, sex

**) simultaneous control of: percentage of single parent households, percentage of 'low-education households'

a) BMI ≥ 90. percentile

b) at least one of the nine check-ups missing

c) at least one out of the nine vaccinations missing

d) German: both parents with mother tongue German; other: all other parents

e) yes: child visited Kindergarten for at least one year; no: all other children

f) percentage of single parent households

g) percentage of persons having the lowest educational level (i.e. 'Hauptschulabschluss')

The results of the multilevel analysis are presented in table 4. The regional variable 'percentage of single parent households' hardly showed any significant associations. Statistical significance was only reached for the dependent variable 'incomplete immunization', and this odds ratio was rather small (0.84 with a CI of 0.74 – 0.95). In order to simplify the presentation, table 4 just shows the results for the model including the regional variable 'percentage of low education households'.

**Table 4:** Multivariate Analysis (multilevel, logistic regression: simultaneous control of all variables)

	overweight^a^		incomplete U1-U9 check-ups^b^		incomplete immunizations^c^	
	OR	95% CI	OR	95% CI	OR	95% CI
individual level						

Mother tongue parents^d^						
- German	1.00		1.00		1.00	
- other	***2.79***	***2.40–3.24***	***2.79***	***2.48–3.13***	***0.63***	***0.57–0.70***
Kindergarten visit of the child^e^						
- yes	1.00		1.00		1.00	
- no	1.12	0.87–1.44	***2.02***	***1.62–2.51***	***1.34***	***1.09–1.63***
Sex of the child						
- boy	1.00		1.00		1.00	
- girl	0.99	0.85–1.14	1.10	0.99–1.23	1.00	0.91–1.09

school district level						

percentage of						
'low education households'^f^						
- low (20.9 – 27.0%)	1.00		1.00		1.00	
- medium (27.1 – 30.8%)	1.23	0.99–1.53	1.06	0.88–1.27	0.92	0.78–1.09
- high (30.9 – 40.9%)	***1.35***	***1.10–1.67***	***1.24***	***1.03–1.48***	***0.78***	***0.66–0.92***

a) BMI ≥ 90. percentile

b) at least one of the nine check-ups missing

c) at least one out of the nine vaccinations missing

d) German: both parents with mother tongue German; other: all other parents

e) yes: child visited Kindergarten for at least one year; no: all other children

f) percentage of persons having the lowest educational level (i.e. 'Hauptschulabschluss')

Concerning the three individual level social variables, the odds ratios in table 4 are very similar to the corresponding odds ratios in table 3, indicating that the additional inclusion of the regional variable 'percentage of low education households' has a small impact. Looking at the odds ratios for this regional variable, though, they are considerably smaller in table 4 than in table 3, and for the group 'high percentage' they are still significant for all three dependent variables. Also, the dose-response pattern can be seen here again.

## Discussion

The analyses clearly point to an association between the mother tongue of the parents on one hand and health risks of their children on the other: For parents with a non-German mother tongue (as compared with German mother tongue parents) the risks of overweight and missing health check-ups are considerably higher, and the risk of missing vaccinations is considerably smaller. A very similar picture emerges when the school districts are characterised by the average educational level: In lower status school districts (as compared with the higher status districts) the risks of overweight and missing health check-ups is higher, and the risk of missing vaccinations is smaller. These associations on the individual level (e.g. mother tongue of the parents) and on the regional level (i.e. average educational level of the school district) are maintained in the multilevel analyses.

The association between migration and health related factors is well known in public health research. Generally speaking, three groups of influencing factors can be distinguished, the psychosocial impact of the migration process, the fact that many migrants live in disadvantaged conditions, and language barriers that hinder the utilization of social and health services. These factors are relevant for adults and for children and they are often discussed in Germany as well [21].

Some studies have already looked at social differences in overweight, participation in the 'U1 to U9' health check-up and vaccinations among children in Germany. The two major public health studies focusing on children have been mentioned already in the introduction, i.e. the HBSC- and the KIGGS-study. The HBSC study shows that the prevalence of most health risks (e.g. smoking, physical inactivity, obesity) is particularly high in the low status group [6], but it focuses on the age group between 11 and 15 years. The KIGGS-study confirms, for example, that problems of missing health check-ups are most prevalent in the low status group, and that there is no clear association between social status and immunization. This study comprises the age group 0–17 years, though, and it does not include small scale regional variables.

The main hypothesis for explaining the association between social status and incomplete immunization is that there is a lot of scepticism concerning immunization in Germany, and that this scepticism is especially common in the population groups with high educational level. They are more aware of potential side effects and they often question the necessity of specific immunizations [30]. It can be hypothesized that migrants are more willing than non-migrants to accept recommendations from health care professionals. It is not quite clear, though, why this increased willingness is restricted to immunizations and does not include health check-ups.

Two other studies are of particular importance here. In the capital city Berlin, a number of health reports have been published concerning the regional and social distribution of health risks among children. These reports include analyses from the medical check-up prior to school enrolment, with the latest report focussing on data from 2005 [31]. In Germany, different states (i.e. 'Laender') have different regulations concerning these medical check-ups, and in Berlin (contrary to Munich, Bavaria) the educational level and employment status of the parents is recorded as well. Based on this information, a simple index is calculated expressing low, medium or high social status of the family. Also, the 'regional socio-economic status' of the 12 districts in Berlin is assessed by combining different information gathered from the statistical office. The results show, for example, that for most health problems (e.g. concerning teeth, missing health check-ups, parental smoking) the prevalence is particularly high in the low status families and the low status districts. Unfortunately, though, these analyses are not advanced to publications in scientific journals, and multilevel analyses are not included.

In another recent study, eight paediatricians have been asked about social barriers concerning utilization of the 'U1 to U9' health check-ups. The study also includes a survey among 644 parents presenting their children to the medical check-up prior to school enrolment [32]. The results of the first part can be summarized in the following way: The paediatricians are very aware of the fact that social problems in the family (e.g. single parent, non-German background, low social status) are associated with increased health risks for the children, and that these social problems can lead to low utilization of the health check-ups. They also stress that the health care financing system does not allow them to spend too much time on helping and advising these families. The results of the second part can be summarized in the following way: Low social status is associated with low utilization of health check-ups. Asked about the reasons for not presenting their child to these health check-ups, the parents stress reasons such as: "I just forgot the check-up" or "My child didn't have any health problem that I couldn't treat myself". It is hypothesized that this kind of answers disguises fundamental problems of health awareness and barriers towards health care utilization.

## Conclusion

Our analyses can add some information to the current discussion, mainly by focussing on a prosperous city such as Munich, by applying very small regional units (i.e. school district), and by including a multilevel analysis. The data from the medical check-up prior to school enrolment provide an excellent basis for public health research in Germany, as response bias is avoided by including the total age cohort. There are some drawbacks that need to be addressed, though: The dataset includes few social indicators for each child (resp. family). It would be important to know more about the social status, for example, but in Munich (as in just about all communities in Germany) this information is not gathered at the medical check-up prior to school enrolment. Also, the dataset includes few indicators on health and health risks for each child that are assessed in a rather objective way. It would be important to add a careful anamnesis and medical examination. Last but not least, no information on environmental factors could be included here for the regional units, such as traffic noise, air pollution, and the availability of parks and playgrounds. It should also be stressed that the classification of overweight is based on the definition 'above the 90-Percent limit', taking the limits proposed for Germany by Kromeyer-Hausschild et al [25]. In international studies overweight is defined, for example, by the method proposed by Cole et al. [26]. We have repeated the analyses with this definition (results not presented here), and we have found very similar results.

Concerning future research on child health, the objective should be to include more information on individual and regional social characteristics on one hand, and on health, health risks and health resources on the other. Also, life course analyses should look at the long term effects of these risks and resources. Concerning public health policy, the major conclusion is that interventions aimed at reducing overweight and increasing the utilization of health check-ups should focus on specific population groups (i.e. parents with a non-German mother tongue) and on top of that on specific regional areas (i.e. school districts with a low social status). Concerning missing vaccinations, though, the message is quite different, as this problem is less prevalent for parents with a non-German mother tongue and in low status school districts. The pros and cons of vaccinating children are hotly disputed in Germany, and many middle and upper class parents oppose it. Efforts to increase immunization should therefore focus on these parents. It is also important to note that utilization of health check-ups and of vaccinations is lower for those children who had not been in a Kindergarten. This could be one more public health reason for promoting Kindergarten visits in Germany.

## Competing interests

The authors declare that they have no competing interests.

## Authors' contributions

DK carried out the statistical analysis and drafted the manuscript. AM contributed the scientific background and supervised the analysis.

## Pre-publication history

The pre-publication history for this paper can be accessed here:


